# Reply to comments from Paz and Shinfeld to article entitled ‘Safety and efficacy of a device to narrow the coronary sinus for the treatment of refractory angina: a single-centre real-world experience’

**DOI:** 10.1007/s12471-016-0904-9

**Published:** 2016-10-26

**Authors:** P. Agostoni, M. Abawi

**Affiliations:** 1 Department of Cardiology, St. Antonius Hospital Nieuwegein, Nieuwegein, The Netherlands; 2 Department of Cardiology, University Medical Center Utrecht, Utrecht, The Netherlands

We welcome the comments of Dr. Paz and Dr. Shinfeld to our article entitled ‘Safety and efficacy of a device to narrow the coronary sinus for the treatment of refractory angina: A single-centre real-world experience’ [1].

We agree with them that the Beck-II [2] procedure has little in common with the coronary sinus reducer as the first is a surgical operation while the second is a percutaneous intervention. However, the concept of narrowing the coronary sinus is similar and this is what we believe makes the two procedures conceptually comparable (interestingly both procedures aim at a residual lumen diameter in the coronary sinus around 3 mm).

We are also pleased to read the personal experience of the colleagues with the coronary sinus reducer. Specifically the possible physio-pathological explanation of the mechanism by which the coronary sinus reducer can exert its function (neovascularisation) is appealing. We only regret we do not have the possibility to read the original data of their preclinical studies as we could not find any pertinent publication with the data briefly mentioned by the colleagues in their letter.
